# Relationship Between Systemic Inflammatory Markers and Histopathological Parameters in Endometrial Adenocarcinoma

**DOI:** 10.3390/jcm15103840

**Published:** 2026-05-16

**Authors:** Özgecan Gündoğar, Sibel Bektaş, Nilgün Bireroğlu, Süleyman Salman, Deniz Buksur, Fatih İrice, Selçuk Cin

**Affiliations:** 1 Department of Pathology, Gaziosmanpasa Training and Research Hospital, University of Health Sciences, Istanbul 34250, Turkey; sibel_bektas@yahoo.com (S.B.); drdenizbuksur@gmail.com (D.B.); 2 Department of Biochemistry, Gaziosmanpaşa Training and Research Hospital, University of Health Sciences, Istanbul 34250, Turkey; nilbireroglu@gmail.com; 3 Department of Obstetrics and Gynaecology, Gaziosmanpaşa Training and Research Hospital, University of Health Sciences, Istanbul 34250, Turkey; sleymansalman@gmail.com (S.S.); fatihirice@gmail.com (F.İ.); 4 Department of Pathology, Cerrahpaşa Faculty of Medicine, Istanbul University-Cerrahpaşa, Istanbul 34320, Turkey; selc2049@hotmail.com

**Keywords:** endometrial cancer, SII, CRP, NLR, survival

## Abstract

**Background/Objectives:** This study aimed to investigate the prognostic value of preoperative systemic inflammatory markers, including the neutrophil-to-lymphocyte ratio (NLR), platelet-to-lymphocyte ratio (PLR), systemic immune–inflammation index (SII), and prognostic nutritional index (PNI), in conjunction with tumor markers and histopathological parameters, on overall survival (OS) and disease-free survival (DFS) in patients with endometrioid adenocarcinoma. Systemic inflammation has been increasingly implicated in tumor progression, and inflammation-based indices derived from routine blood tests offer a practical approach for preoperative risk assessment. **Methods**: A total of 155 patients diagnosed with endometrioid adenocarcinoma between 2016 and 2025 were retrospectively analyzed. The NLR, PLR, SII, and PNI were calculated from preoperative complete blood count and biochemical data. Associations with histopathological parameters, including FIGO grade, myometrial invasion, cervical stromal involvement, lymphovascular invasion, and lymph node metastasis, were evaluated. Survival analyses were performed using the Kaplan–Meier method and Cox proportional hazards regression models. **Results**: Patients with cervical stromal involvement had significantly higher NLR values (*p* = 0.028) and lower lymphocyte counts (*p* = 0.032). Additionally, lower albumin and PNI levels were observed in patients with cervical stromal involvement (*p* = 0.034 and *p* = 0.031, respectively). Multivariate Cox regression analysis identified advanced age (HR: 1.09, *p* < 0.001), subtotal hysterectomy (HR: 3.76, *p* = 0.006), lymph node metastasis (HR: 4.78, *p* = 0.020), elevated CRP levels (HR: 1.05, *p* = 0.004), high SII (HR: 1.002, *p* = 0.037), and increased CA 15-3 levels (HR: 1.04, *p* < 0.001) as independent predictors of poor OS. CA-125 level was an independent risk factor for DFS (*p* = 0.031). **Conclusions**: Systemic inflammatory markers, particularly SII and NLR, may serve as useful prognostic indicators for endometrioid adenocarcinoma. Their association with survival outcomes and local tumor extension highlights their potential clinical value in preoperative risk stratification and individualized treatment planning. Future prospective studies integrating these markers with molecular classification data are required.

## 1. Introduction

Endometrial cancer is one of the most common gynecological malignancies worldwide, with an estimated incidence that has been rising over recent decades, largely attributed to the increasing rates of obesity and metabolic syndrome [1]. Endometrioid adenocarcinoma accounts for most cases and is typically diagnosed at an early stage, contributing to a generally favorable prognosis. However, prognosis is largely determined by histopathological risk factors, such as the FIGO stage, histological grade, depth of myometrial invasion, lymphovascular space invasion, cervical stromal involvement, and lymph node metastasis [1,2]. Despite advances in surgical and adjuvant treatment strategies, a subset of patients with apparently low-risk disease experiences recurrence, underscoring the need for more reliable and accessible preoperative risk stratification tools.

The tumor microenvironment and systemic host responses play critical roles in cancer progression. Systemic inflammation has been increasingly implicated in tumor growth, invasion, and metastasis. Neutrophils promote angiogenesis and tissue invasion by secreting vascular endothelial growth factor (VEGF) and matrix metalloproteinase (MMPs). Platelets facilitate tumor cell dissemination by forming protective microthrombi in the circulation, whereas lymphocytes are the principal mediators of antitumor immunity [3,4]. The balance between these pro- and antitumor components can be captured using simple hematological indices derived from routine preoperative blood tests.

Accordingly, inflammation-based indices, including the neutrophil-to-lymphocyte ratio (NLR), platelet-to-lymphocyte ratio (PLR), systemic immune–inflammation index (SII = platelet × neutrophil/lymphocyte), and prognostic nutritional index (PNI = albumin × 10 + lymphocyte × 0.005), have been proposed as practical, cost-effective, and reproducible biomarkers of tumor–host interaction [4,5]. These markers are readily available from standard preoperative laboratory assessments and do not require additional costs or invasive procedures, making them particularly attractive for clinical implementation. Previous studies have associated higher NLR, PLR, and SII and lower PNI with poorer disease-free and overall survival in various malignancies, including endometrial cancer [4,6,7,8,9].

Recent evidence supports the prognostic utility of these markers across different cancer types and clinical contexts. Tur-Martínez et al. demonstrated that preoperative inflammatory and nutritional biomarkers were independently associated with survival outcomes in patients undergoing gastrectomy for gastric adenocarcinoma, highlighting the broader applicability of these indices to solid tumors [10]. Uzun et al. emphasized the relationship between nutritional status and systemic immune–inflammation indices, underscoring the composite nature of PNI as a marker that integrates both inflammatory and nutritional dimensions [8]. In the context of gynecological oncology, Alci et al. reported significant associations between peripheral inflammatory markers and disease severity in cervical pathology, suggesting a shared inflammatory mechanism across gynecological malignancies [9].

In addition to systemic inflammatory indices, several serum tumor markers, including carcinoembryonic antigen (CEA), cancer antigen 19-9 (CA 19-9), CA-125, CA 15-3, and alpha-fetoprotein (AFP), were incorporated into the study. These markers are routinely assessed as part of the preoperative workup in patients with suspected or confirmed gynecological malignancies at our institution, and their preoperative values were therefore available for all patients. Furthermore, given that both inflammatory and tumor-derived signals may jointly shape the tumor microenvironment and influence disease behavior, we aimed to investigate whether these markers carry independent or combined prognostic value alongside the inflammatory indices, thereby providing a more comprehensive preoperative biomarker profile.

In this study, we investigated the relationships between preoperative systemic inflammatory markers and key histopathological parameters, including FIGO grade, depth of myometrial invasion, cervical stromal invasion, lymphovascular and perineural invasion, and lymph node involvement, as well as overall survival (OS) and disease-free survival (DFS) in patients with endometrioid adenocarcinoma. We hypothesized that elevated inflammatory indices and reduced PNI are associated with adverse pathological features and worse survival outcomes, thereby contributing to more individualized preoperative risk stratification.

## 2. Materials and Methods

### 2.1. Study Design and Population

This single-center retrospective study included patients diagnosed with endometrioid adenocarcinoma and treated surgically at Gaziosmanpaşa Training and Research Hospital between 2016 and 2025. Among the 200 initially screened patients with available preoperative complete blood count data, 15 were excluded due to unavailable pathology materials, 20 due to missing preoperative blood tests, and 10 due to incomplete disease-free survival (DFS) or overall survival (OS) data. The final analysis included 155 patients. 

The inclusion criteria were as follows:

(1) Histologically confirmed endometrioid adenocarcinoma;
(2) Availability of complete preoperative hematological and biochemical data;
(3) Availability of complete follow-up data.

The exclusion criteria were as follows:

(1) Receipt of neoadjuvant therapy prior to surgery;
(2) Presence of synchronous malignancies;
(3) Active infectious or autoimmune diseases at the time of blood sampling;
(4) Incomplete pathological or survival data.

All pathology slides were re-evaluated by two experienced pathologists using the standardized World Health Organization criteria.

### 2.2. Follow-Up

Postoperative follow-up was conducted at regular intervals: every 3 months for the first 2 years, every 6 months for years 3–5, and annually thereafter until the present. Overall survival (OS) was defined as the time from surgery to death from any cause or the last follow-up. Disease-free survival (DFS) was defined as the time from surgery to the first documented recurrence or death, whichever occurred first.

### 2.3. Data Collection and Definition of Inflammatory Markers

Preoperative hematological data were obtained from the hospital’s laboratory information system. Blood samples were collected within one week before surgery. The systemic inflammatory markers were calculated as follows:

NLR: neutrophil count/lymphocyte count [11];
PLR: platelet count/lymphocyte count [11];
SII: (platelet count × neutrophil count)/lymphocyte count [12];
PNI: albumin (g/dL) × 10 + lymphocyte count (cells/mm^3^) × 0.005 [13].

### 2.4. Histopathological Evaluation

Resection specimens were reviewed according to the World Health Organization criteria by two experienced pathologists blinded to the clinical outcomes. The recorded parameters included histological type, FIGO grade, depth of myometrial invasion, cervical stromal invasion, lymphovascular invasion, perineural invasion, and lymph node metastasis [14,15]. Myometrial invasion was categorized as less than 50% or ≥50%.

### 2.5. Statistical Analysis

Normality was assessed using the Shapiro–Wilk test. Group comparisons were performed using the independent-samples *t*-test or Mann–Whitney U test and the Kruskal–Wallis test with Dunn’s post hoc analysis for multiple groups. Categorical variables were analyzed using the chi-square test. OS and DFS were evaluated using the Kaplan–Meier method with log-rank tests. Independent prognostic factors were identified using Cox proportional hazards regression with the backward likelihood-ratio method, with an entry criterion of *p* < 0.05 and a removal criterion of *p* > 0.10. All tumor markers (CEA, CA 19-9, CA-125, CA 15-3, and AFP) were also analyzed as continuous variables, consistent with the approach applied to the inflammatory markers. No universally accepted cutoff values exist for the evaluated inflammatory markers; therefore, continuous values were used in all analyses. Continuous variables are expressed as mean ± standard deviation or median (minimum–maximum), and categorical variables are expressed as frequencies and percentages. Statistical analyses were performed using IBM SPSS Statistics version 29.0.2 (IBM Corp., Armonk, NY, USA); *p*-values < 0.05 were considered statistically significant.

## 3. Results

A total of 155 patients with endometrioid adenocarcinoma were included in the study. The mean age was 60.13 ± 9.68 years, and the mean tumor size was 4.26 ± 4.12 cm. Mean DFS and OS were 70.99 ± 29.88 and 71.85 ± 29.22 months, respectively. Total hysterectomy was performed in 148 (95.5%) patients, and a subtotal hysterectomy was performed in seven (4.5%) patients. FIGO grade 1 was present in 32.9%, grade 2 in 50.3%, and grade 3 in 16.8% of patients (Figures S1 and S2). Lymphovascular invasion was present in 53.5% of patients (Figure S3), perineural invasion in 6.5%, cervical stromal involvement in 14.8%, and lymph node metastasis in 5.8%. At the time of analysis, 23.2% of the patients had died, and 7.1% had experienced recurrence.

Most systemic inflammatory markers showed no significant correlation with age; only CEA levels increased significantly with advancing age (r = 0.272, *p* < 0.001). Inflammatory indices (NLR, PLR, and SII), albumin, PNI, and blood cell counts did not differ according to the surgical type, whereas CA 19-9 levels were higher in patients who underwent total hysterectomy (*p* = 0.031).

Tumor size was not associated with NLR, PLR, SII, albumin, or PNI, but correlated positively with CA 19-9 (r = 0.183, *p* = 0.027) (Table 1). FIGO grade was not associated with NLR, PLR, SII, albumin, PNI, or blood cell counts. CA 19-9 levels were higher in patients with lymphovascular invasion (*p* = 0.040), whereas other markers showed no differences. No significant associations were observed between perineural invasion and depth of myometrial invasion.

Patients with cervical stromal involvement had significantly higher NLR (*p* = 0.028) and lower lymphocyte counts (*p* = 0.032) (Table 2). No differences were found according to endocervical involvement. CA 15-3 levels were higher in patients with lymph node involvement (*p* = 0.012), and AFP levels were higher in those without lymph node metastasis (*p* = 0.006).

Deceased patients were significantly older, had shorter DFS and OS, and were more likely to have undergone subtotal hysterectomy (*p* < 0.05).

**Figure 1:**
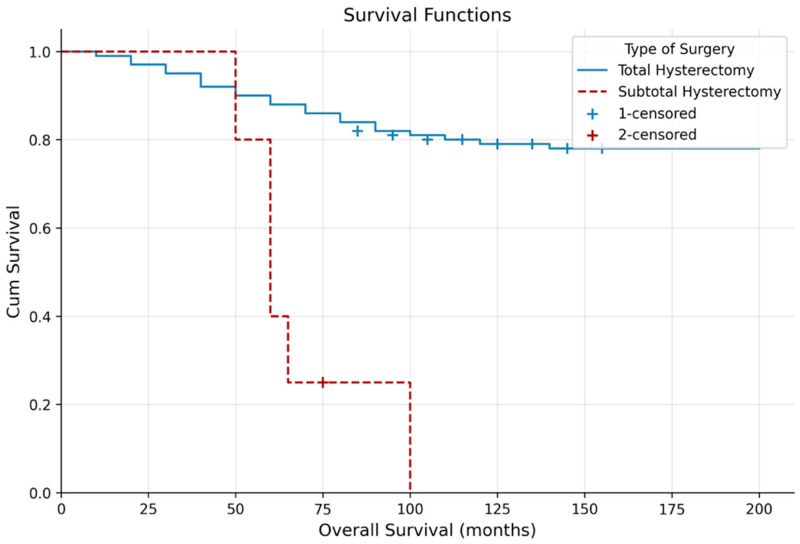
Kaplan–Meier curve of overall survival according to type of surgery.

**Figure 2:**
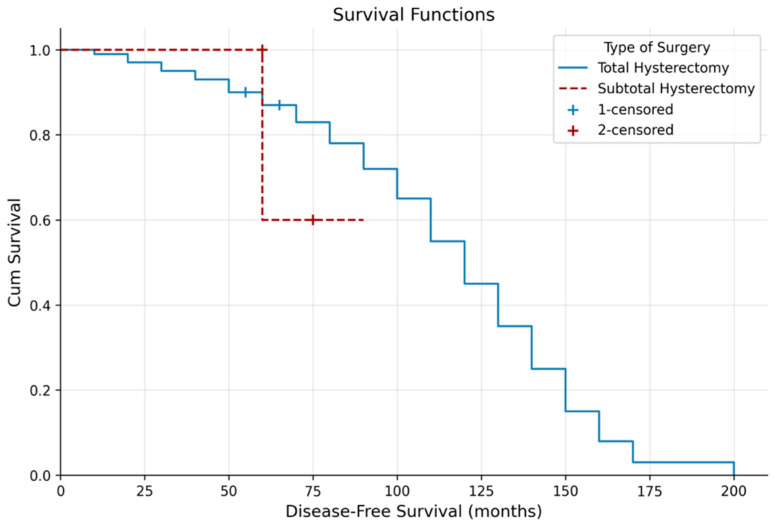
Kaplan–Meier curve of disease-free survival according to type of surgery.

Kaplan–Meier analysis showed significantly longer OS in patients who underwent total hysterectomy compared with subtotal hysterectomy (133.25 ± 4.40 vs. 62.61 ± 7.81 months; *p* = 0.003), with no difference in DFS (*p* = 0.909) (Table 3, Figure 1 and Figure 2). Lymph node metastasis was associated with shorter OS (*p* = 0.036) but not DFS (*p* = 0.605).

In multivariate Cox regression analysis for OS, advanced age (HR = 1.098, *p* < 0.001), subtotal hysterectomy (HR = 3.769, *p* = 0.006), lymph node metastasis (HR = 4.782, *p* = 0.020), elevated CRP (HR = 1.052, *p* = 0.004), high SII (HR = 1.002, *p* = 0.037), and increased CA 15-3 (HR = 1.048, *p* < 0.001) were independent adverse prognostic factors (Table 4).

For DFS, neutrophil count was independently protective (HR = 0.831, *p* = 0.004), whereas higher lymphocyte count (HR = 1.780, *p* = 0.001) and CA-125 level (HR = 1.005, *p* = 0.031) were independent risk factors; age showed borderline significance (*p* = 0.057). Other variables were not independently associated with DFS (Table 5).

ROC analyses demonstrated that none of the evaluated clinical or laboratory parameters significantly discriminated between surviving and deceased patients (all *p* > 0.05). This apparent discrepancy between the Cox regression and ROC analysis results warrants clarification. Cox proportional hazards regression identifies variables that are independently associated with survival time, answering the question of whether a variable is a significant risk factor after controlling for confounders. ROC analysis, on the other hand, evaluates the ability of a variable to discriminate between two binary outcomes (e.g., deceased vs. alive) at a single time point, without accounting for survival time or competing variables. Therefore, a variable may be a statistically significant prognostic factor in Cox regression while simultaneously demonstrating limited discriminatory power in ROC analysis. In our study, this pattern likely reflects the fact that the evaluated markers contribute to survival prediction in a multivariable context but lack sufficient standalone discriminatory ability to separate survivors from non-survivors as individual tests.

## 4. Discussion

Endometrioid adenocarcinoma is often detected at an early stage; however, disease management remains challenging in cases with aggressive histopathological features. The biological behavior of endometrioid adenocarcinoma is determined by the complex interaction between tumor-related characteristics and the systemic response of the patient to the disease. This study aimed to elucidate the prognostic value of systemic inflammatory markers obtained from routine preoperative blood tests across the clinicopathological spectrum of endometrioid adenocarcinoma, thereby providing a cost-effective contribution to preoperative risk stratification.

In our study, CEA levels increased significantly with age. CA 19-9 levels were significantly higher in patients who underwent total hysterectomy than in those who underwent subtotal hysterectomy. No significant correlation was identified between albumin or PNI levels and tumor size or the FIGO histological grade. Notably, albumin and PNI levels were significantly lower in patients with cervical stromal involvement (*p* = 0.034 and *p* = 0.031, respectively), suggesting that a poorer nutritional status may be associated with local tumor extension. Furthermore, the significant association between lower albumin and PNI levels and cervical stromal involvement suggests that nutritional status may play a role in local tumor aggressiveness. This finding highlights the potential value of incorporating nutritional indices and inflammatory markers in preoperative risk assessment. NLR values were significantly higher in patients with cervical stromal involvement, whereas lymphocyte counts were significantly lower in the presence of cervical involvement. CA 15-3 levels were significantly lower in patients without lymph node involvement than in those with lymph node involvement, whereas AFP levels were significantly higher in patients without lymph node metastasis. Overall survival was significantly longer in patients without lymph node metastases. Furthermore, age, type of surgery, lymph node metastasis, and particularly the systemic inflammatory markers CRP and SII, were identified as independent prognostic factors for overall survival in endometrial adenocarcinoma. With respect to disease-free survival, neutrophil and lymphocyte levels were found to be especially determinant, indicating that DFS is more closely associated with direct cellular parameters rather than with conventional ratio-based indices, such as NLR or PLR. Overall, although inflammation-based markers alone do not explain all clinical outcomes, our findings suggest that when considered in conjunction with appropriate clinicopathological variables, they can contribute meaningfully to survival prediction and serve a supportive role in risk stratification. The independent protective effect of peripheral neutrophil count on DFS (HR = 0.831, *p* = 0.004), however, does not directly align with the pro-tumoral functions attributed to neutrophils in the literature, and this discrepancy may be explained by the functional distinction between tumor-associated neutrophils (TANs) and circulating peripheral neutrophils. TANs recruited and polarized within the tumor microenvironment predominantly adopt a pro-tumoral phenotype (N2) that promotes angiogenesis, tissue invasion, and immune evasion [16]. In contrast, peripheral blood neutrophils retain a distinct functional profile and may reflect a competent systemic innate immune response against tumor dissemination [17]. Several studies have demonstrated that peripheral neutrophil counts do not necessarily mirror the behavior of TANs and may independently contribute to antitumoral surveillance [18]. Therefore, the protective association observed in our study may reflect the systemic innate immune competence of the host rather than direct pro-tumoral activity.

NLR is one of the most fundamental parameters reflecting the balance between pro-tumoral inflammation mediated by neutrophils and antitumor immunity mediated by lymphocytes. In our study, the significantly higher NLR observed in patients with cervical stromal involvement (*p* = 0.028) indicates that this ratio is directly associated with the tumor’s potential to extend beyond the uterine confines and infiltrate adjacent tissues, such as the cervical stroma. Similarly, Ni et al. reported a strong correlation between elevated preoperative NLR and higher FIGO histological grade and deep myometrial invasion [11]. From a biological perspective, this phenomenon can be explained by the ability of neutrophils to promote angiogenesis and tissue invasion within the tumor microenvironment through the secretion of vascular endothelial growth factor (VEGF) and matrix metalloproteinase (MMPs). The strong association between NLR and cervical involvement observed in our study supports the potential use of this parameter as a preoperative indicator of local tumor spread for risk stratification. However, in contrast to some recent prospective studies, such as that by Njoku et al., NLR was not identified as an independent prognostic factor for survival in our cohort, suggesting that this marker may be more sensitive in predicting the anatomical pattern of tumor spread rather than long-term survival outcomes [2].

Platelets function as protective escorts for cancer cells in the circulatory system. The platelet-to-lymphocyte ratio (PLR) represents the relationship between protective mechanisms and immune resistance. In our study, the borderline significant trend observed between PLR and lymph node involvement (*p* = 0.075) is consistent with the hypothesis that platelet-dominant inflammation facilitates metastatic spread [11,19]. Tumor cells aggregate with platelets to form a microthrombus layer, which shields them from immune surveillance and enhances their adhesion to the vascular endothelium, thereby promoting extravasation. Ni et al. demonstrated that elevated PLR values were strongly associated with poor overall survival in endometrioid adenocarcinoma and that platelet activity increased in advanced-stage diseases [11]. Similarly, Doğan et al. reported that high preoperative platelet counts were associated with disease extent and metastatic potential [19]. In our cohort, the higher PLR observed in patients with lymph node metastasis supports the hypothesis that platelets facilitate tumor cell adhesion to the vascular endothelium and promote infiltration into lymphatic channels [2]. The finding that PLR alone was not as dominant as SII in predicting survival outcomes indicates that, although the coagulation system is an important component of cancer progression, it yields a clearer prognostic signal when combined with neutrophil-driven inflammatory responses. The observed trend toward lymph node involvement further supports the role of platelets in metastasis.

SII is a more comprehensive biomarker than NLR and PLR because it integrates inflammation, coagulation, and immune suppression into a single formula. However, it should be noted that although SII reached statistical significance as an independent prognostic factor (HR = 1.002, *p* = 0.037), the absolute magnitude of this hazard ratio was very close to unity, indicating a minimal per-unit increase in risk. This suggests that while the SII carries statistically demonstrable prognostic information, its clinical impact at the individual patient level may be modest. Therefore, the clinical utility of the SII should be considered in the context of its composite nature, integrating platelet, neutrophil, and lymphocyte counts, rather than its hazard ratio alone. Clinicians may find greater value in using the SII as part of a multiparametric risk panel rather than as a standalone predictor. The most striking finding of our study was the identification of SII as an independent risk factor for overall survival (*p* = 0.037). Because the SII simultaneously reflects inflammatory status, coagulation activity, and immune dysfunction, it provides a more integrated assessment of tumor–host interactions. The identification of SII as an independent prognostic factor for overall survival in our Cox regression analysis represents one of the most critical findings of our study, highlighting the superiority of this index in endometrioid adenocarcinoma.

Huang et al. reported that SII is a superior prognostic factor compared to NLR and PLR in predicting survival in endometrioid adenocarcinoma [20]. An increase in SII creates a pro-tumor microenvironment that facilitates the entry of tumor cells into the circulatory system. The association between elevated SII and increased mortality risk indicates that the numerical dominance of pro-tumoral cells (neutrophils and platelets) over lymphocytes directly worsens the disease progression. Lei et al. further emphasized that SII is critical in predicting lymph node metastasis; in our study, the direct impact of SII on survival likely reflects the ultimate effect of this metastatic potential on the patient outcomes [21]. Therefore, SII represents not only the anatomical spread of the tumor but also a powerful systemic index indicating the patient’s diminished ability to counteract this biological crisis. Thus, it can be considered a more reliable preoperative biomarker than NLR and PLR for estimating survival expectancy.

The prognostic nutritional index (PNI) reflects both the inflammatory and nutritional dimensions of tumor–host interaction, as it incorporates both albumin levels and lymphocyte counts. In our study, lower PNI levels were significantly associated with cervical stromal involvement (*p* = 0.031), suggesting that impaired nutritional status may contribute to local tumor aggressiveness. This finding is consistent with previous reports demonstrating that a reduced PNI is associated with adverse pathological features and poorer survival in gynecological malignancies. Albumin, as a component of PNI, reflects not only nutritional status but also systemic inflammatory burden, as it is a negative acute phase reactant. Therefore, the association between a lower PNI and cervical stromal involvement may reflect the combined effects of malnutrition and heightened systemic inflammation in facilitating tumor invasion. Future studies should further explore the independent contribution of PNI to survival outcomes in endometrioid adenocarcinoma, particularly in the context of molecular classification. In our study, elevated CRP levels significantly increased the risk of mortality (HR = 1.052, *p* = 0.004). This finding supports the observation emphasized by Njoku et al. that the preoperative systemic inflammatory burden is directly associated with oncological outcomes [2]. The identification of CA 15-3 as an independent adverse prognostic factor for OS (HR = 1.048, *p* < 0.001) represents one of the most distinctive findings of our study. Although CA 15-3 is primarily recognized as a breast cancer tumor marker, its prognostic significance in endometrial cancer has been previously reported. In a study analyzing multiple serum tumor markers in 97 patients with endometrial cancer, elevated CA 15-3 was significantly associated with poor prognostic clinical factors, and in multivariate analysis, CA 15-3 was the most significant independent prognostic marker with the largest hazard ratio [22]. Furthermore, CA 15-3 is an epitope of the MUC1 glycoprotein, which is overexpressed in approximately 86% of endometrial tumors and is associated with increased cellular proliferation, tumor invasion, and poor prognosis [23]. Similarly, the independent association of CA-125 with DFS (HR = 1.005, *p* = 0.031) is consistent with the broader literature. A comprehensive meta-analysis of 25 studies involving 7716 patients demonstrated that elevated preoperative CA-125 levels are significantly associated with poorer OS and DFS in endometrial cancer, likely reflecting an increased propensity for lymph node metastasis and peritoneal dissemination [24].

This study had several methodological limitations. The retrospective design represents an inherent limitation, as it precludes full control over exogenous factors that may influence systemic inflammation, such as body mass index (obesity), diabetes mellitus, and smoking.

The relatively small number of patients in certain histopathological subgroups (such as those with lymph node metastasis, perineural invasion, and disease recurrence) may have limited the statistical power to demonstrate the associations between these variables and systemic inflammatory markers. Additionally, the lack of universally accepted standard cutoff values for inflammatory and tumor markers restricted the determination of optimal thresholds in this study. Finally, because molecular classification data were not available, it was not possible to comprehensively evaluate the relationship between systemic inflammation and tumor molecular biology. Therefore, the findings of this study should be interpreted within the context of the clinicopathological and biochemical parameters. Furthermore, although body mass index, diabetes mellitus, and smoking status were acknowledged as potential confounders, these variables were not systematically recorded and could not be included in regression models. These factors are known to independently influence neutrophil, lymphocyte, and platelet counts, and their absence may have introduced unmeasured confounding factors into the inflammatory marker analyses. Future prospective studies should incorporate these variables to better isolate the tumor-specific contribution of inflammatory indices to prognosis. Additionally, the relatively long inclusion period (2016–2025) may have introduced heterogeneity in clinical management, as surgical techniques, adjuvant therapy protocols, and postoperative follow-up practices may have evolved during this period. It should be noted that data collection was completed in 2025, and the manuscript was submitted in 2026; therefore, the inclusion of patients treated in 2025 does not represent future data. Although all pathology slides were re-evaluated by experienced pathologists using standardized criteria, the potential impact of temporal changes in clinical practice on study outcomes cannot be fully excluded. Furthermore, the prognostic models developed in this study were not subjected to internal (e.g., bootstrapping) or external validation in an independent cohort, which limits the generalizability of the findings. Validation in larger, prospective, or multicenter studies is warranted before these models can be applied in routine clinical practice. Multiple comparisons were performed without correction for type I errors, which may have increased the risk of false-positive findings. This represents an additional methodological limitation, and the results should be interpreted accordingly in future studies. Additionally, the Cox regression models included a relatively large number of variables in relation to the number of observed events, which may have increased the risk of overfitting the model. Therefore, the results of the multivariate analyses should be interpreted with caution, and replication in larger cohorts is recommended. Finally, no universally accepted cutoff values exist for NLR, PLR, SII, or PNI in endometrial cancer, and optimal thresholds could not be determined in this study because of the lack of significant ROC discrimination. This limits the direct clinical applicability of these markers and highlights the need for standardized cutoff values in future studies. Additionally, molecular classification data (e.g., TCGA subgroups: POLE-mutated, mismatch repair-deficient, p53-abnormal, and no specific molecular profile) were not available in this retrospective cohort, as routine molecular profiling was not performed at our institution during the study period. Given that molecular classification is increasingly integrated into endometrial cancer management and prognostic stratification, its absence represents a significant limitation of this study. Future studies should incorporate molecular classification and systemic inflammatory markers to develop more comprehensive and clinically applicable prognostic models.

These findings indicate that systemic inflammatory markers are not merely auxiliary tools but fundamental components in the management of endometrioid adenocarcinoma. While NLR primarily reflects local anatomical aggressiveness, such as cervical involvement, SII serves as a predictor of long-term oncological survival. Moreover, the survival advantage of total hysterectomy over subtotal procedures (*p* = 0.003) in oncologic surgery confirms surgical radicality as one of the most decisive factors influencing prognosis.

In the future, combining these markers with molecular parameters such as vascular endothelial growth factor (VEGF) expression, as discussed by Zinovkin et al., may add a new dimension to the “personalized treatment” approach through the use of preoperative blood-based biomarkers [25]. This perspective highlights the need for comprehensive models that integrate inflammatory markers with conventional clinicopathological variables to improve prognostic accuracy [2,4]. Accordingly, future research should focus on the development of prognostic models that combine systemic inflammatory markers and clinicopathological parameters to enable more precise and accurate risk stratification in patients diagnosed with endometrioid adenocarcinoma [2].

## 5. Conclusions

In conclusion, this study demonstrates that preoperative systemic inflammatory markers, particularly SII and NLR, may serve as useful prognostic indicators in endometrioid adenocarcinoma. SII was identified as an independent predictor of overall survival, while NLR was significantly associated with cervical stromal involvement, suggesting its role in reflecting local tumor aggressiveness. Additionally, lower albumin and PNI levels in patients with cervical stromal involvement highlight the potential contribution of nutritional status to tumor progression. Although the clinical effect size of SII was modest, its composite nature, integrating platelet, neutrophil, and lymphocyte counts, makes it a practical and cost-effective tool for preoperative risk stratification. These findings should be interpreted in light of the study’s retrospective design and the methodological limitations outlined above.

From a clinical perspective, since markers such as SII, CRP, CA 15-3, and CA-125 can be obtained from routine preoperative blood tests without any additional cost, their incorporation alongside existing clinicopathological parameters may practically contribute to the identification of high-risk patients preoperatively, the planning of the extent of surgical staging, and the individualization of adjuvant therapy decisions. Furthermore, future studies should focus on developing a predictive nomogram or clinical scoring system that integrates systemic inflammatory markers (SII, NLR), tumor markers (CA 15-3, CA-125), and traditional clinicopathological parameters (age, type of surgery, and lymph node status). Such an integrated tool could strengthen preoperative risk assessment in patients with endometrioid adenocarcinoma and provide clinicians with a readily applicable guide in daily practice. Future prospective multicenter studies incorporating molecular classification data alongside inflammatory markers are needed to validate these findings and develop more comprehensive prognostic models for individualized treatment planning.

## Figures and Tables

**Table 1:** Correlations between tumor size and systemic inflammatory markers and tumor-related laboratory parameters (Spearman correlation analysis).

		Tumor Size
NLR	r	−0.120
	*p*-value	0.138
PLR	r	0.043
	*p*-value	0.592
SII	r	−0.021
	*p*-value	0.798
Albumin	r	−0.061
	*p*-value	0.668
PNI	r	−0.060
	*p*-value	0.674
Neutrophil	r	−0.039
	*p*-value	0.633
Lymphocyte	r	0.065
	*p*-value	0.426
Platelet	r	0.033
	*p*-value	0.688
CEA	r	0.134
	*p*-value	0.107
CA199	r	0.183
	*p*-value	0.027
CA125	r	0.142
	*p*-value	0.087
CA153	r	0.052
	*p*-value	0.532
CRP	r	−0.025
	*p*-value	0.760
AFP	r	−0.016
	*p*-value	0.852

*p*-values were derived from Spearman correlation analysis.

**Table 2:** Comparison of systemic inflammatory markers according to cervical stromal involvement.

Cervical Involvement	Absent	Present	*p*-Value ^c^
NLR	2.26 (1–29.51)	3.15 (0.99–22.74)	**0.028**
PLR	129.51 (1–591.12)	131.21 (71.08–335.71)	0.370
SII	642.07 (145.01–5990.57)	675.36 (241.34–5344.57)	0.314
Albumin	4.00 (1.81–4.89)	3.35 (2.59–3.70)	0.034
PNI	40.01 (18.10–48.92)	33.51 (25.91–37.01)	0.031
Neutrophil	5.05 (1.31–14.46)	5.99 (2.35–15.92)	0.171
Lymphocyte	2.07 (0.49–5.39)	1.66 (0.70–4.15)	**0.032**
Platelet	261.50 (69–507)	235 (158–455)	0.177
CEA	1.70 (0.39–7.73)	1.84 (0.79–6.81)	0.477
CA 199	18 (0–277)	23.60 (0.80–131)	0.563
CA 125	17.82 (4.70–284.90)	21.60 (6.70–261)	0.257
CA 153	13.35 (4–110)	15.42 (7–86.70)	0.191
CRP	7.29 (0–30.80)	8.72 (2–108)	0.260
AFP	3.53 (1.45–12.78)	3.49 (1.33–3.76)	**0.050**

^c^ Comparisons were performed using the Mann–Whitney U test. Data are presented as median (minimum–maximum). Bold values indicate statistical significance (*p* < 0.05).

**Table 3:** Kaplan–Meier analysis according to type of surgery.

	Mean Survival Time			
**Overall survival (months)**	**Estimate**	**Std. Error**	**%95 CI**	***p*-value**
**Type of Surgery**				
Total hysterectomy	133.25	4.40	124.62–141.87	**0.003**
Subtotal hysterectomy	62.61	7.81	47.30–77.93	
**Disease-free survival (months)**	**Estimate**	**Std. Error**	**%95 CI**	***p*-value**
**Type of Surgery**				
Total hysterectomy	81.10	2.46	76.27–85.93	0.909
Subtotal hysterectomy	74.80	6.68	61.70–87.90	

*p*-values were derived from the log-rank test. Bold values indicate statistical significance (*p* < 0.05).

**Table 4:** Multivariate Cox proportional hazards analysis of factors affecting overall survival.

	β	Std. Error	HR	%95 CI	*p*-Value
Age	0.093	0.021	1.098	1.053–1.145	**<0.001**
PN	−2.821	1.217	0.060	0.005–0.647	**0.020**
Type of surgery (ref: total hysterectomy)					
Subtotal Hysterectomy	1.327	0.484	3.769	1.459–9.737	**0.006**
Lymph node metastasis (ref: absent)	1.565	0.674	4.782	1.276–17.917	**0.020**
NLR	−0.349	0.231	0.705	0.448–1.110	0.131
CA153	0.047	0.011	1.048	1.025–1.072	**<0.001**
CRP	0.050	0.018	1.052	1.016–1.088	**0.004**
SII	0.200	0.100	1.221	1.004–1.486	**0.037**
Tumor size	0.024	0.054	1.025	0.921–1.139	0.655
Figo (ref:1)					0.618
Figo 2	0.600	0.619	1.823	0.541–6.137	0.332
Figo 3	0.415	0.864	1.515	0.278–8.244	0.631
LV	0.197	0.613	1.217	0.366–4.046	0.748
Depth of myometrial invasion (ref < %50)	0.211	0.557	1.235	0.414–3.680	0.705
Cervical Involvement (Ref: absent)	0.768	0.851	2.156	0.406–11.432	0.367
Endocervical Involvement (Ref: absent)	−0.741	0.891	0.477	0.083–2.734	0.406
Lymph Node (Ref: absent)	0.162	0.558	1.176	0.394–3.513	0.771
Neutrophil	−0.118	0.404	0.889	0.402–1.963	0.770
Lymphocyte	−0.127	0.710	0.881	0.219–3.541	0.858
Platelet	−0.003	0.009	0.997	0.980–1.014	0.689
PLR	−0.006	0.015	0.994	0.964–1.024	0.683
Albumin	−0.023	0.016	0.978	0.947–1.009	0.164
CA199	0	0.003	1	0.995–1.006	0.956
CA125	−0.006	0.007	0.994	0.981–1.007	0.354
AFP	0.023	0.190	1.024	0.706–1.485	0.902

Overall significance: *p* < 0.001; Model: Backward LR. SII is reported per 100-unit increase. The HR of 1.000 observed for CA 19-9 reflects its lack of independent prognostic significance in this model (*p* = 0.956) rather than a scaling error. Bold values indicate statistical significance (*p* < 0.05).

**Table 5:** Multivariate Cox proportional hazards analysis of factors affecting disease-free survival.

	β	Std. Error	HR	%95 CI	*p*-Value
Age	0.024	0.013	1.025	0.999–1.051	0.057
Perineural invasion	0.120	0.570	1.127	0.369–3.444	0.833
Lymph node metastasis (ref: absent)	0.761	0.527	2.141	0.762–6.011	0.148
NLR	0.188	0.284	1.206	0.692–2.103	0.508
CA153	0.010	0.014	1.010	0.982–1.039	0.485
CRP	0	0.021	1	0.961–1.042	0.982
SII	0	0.002	1	0.997–1.003	0.992
Tumor size	0.009	0.026	1.009	0.960–1.061	0.722
Figo (ref:1)					0.529
Figo 2	0.225	0.264	1.252	0.746–2.100	0.394
Figo 3	0.468	0.432	1.597	0.685–3.724	0.279
Lymphovascular invasion	−0.301	0.288	0.740	0.421–1.301	0.296
Depth of myometrial invasion (ref < %50)	−0.286	0.339	0.751	0.387–1.460	0.399
Cervical Involvement (Ref: absent)	0.856	0.557	2.353	0.789–7.015	0.125
Endocervical Involvement (Ref: absent)	−0.807	0.568	0.446	0.146–1.359	0.156
Lymph Node (Ref: absent)	−0.436	0.253	0.647	0.394–1.062	0.085
Neutrophil	−0.185	0.064	0.831	0.734–0.942	**0.004**
Lymphocyte	0.576	0.178	1.780	1.256–2.522	**0.001**
Platelet	0.003	0.005	1.003	0.993–1.014	0.516
PLR	−0.004	0.008	0.996	0.980–1.012	0.592
Albumin	0.005	0.008	1.005	0.990–1.021	0.494
CA199	−0.001	0.001	0.999	0.997–1.001	0.431
CA125	0.005	0.002	1.005	1–1.1.009	**0.031**
AFP	0.042	0.082	1.043	0.887–1.225	0.612

Overall significance: *p* = 0.008; Model: Backward LR. The HR values of 1.000 observed for SII and CRP reflect their lack of independent prognostic significance in this model (*p* = 0.992 and *p* = 0.982, respectively) rather than a scaling error. Bold values indicate statistical significance (*p* < 0.05).

## Data Availability

The data presented in this study are available upon request from the corresponding author.

## Supplementary Materials

The following supporting information can be downloaded at: https://www.mdpi.com/article/10.3390/jcm15103840/s1, Figure S1: Typical endometrioid glands exhibiting a cribriform architecture within the stromal tissue (H&E ×100); Figure S2: Cribriform glandular structures and squamous differentiation characteristic of endometrioid adenocarcinoma (H&E ×100; Figure S3: Lymphovascular invasion in endometrioid adenocarcinoma (H&E ×100).
