# Supplementary material for: Relationship Between Systemic Inflammatory Markers and Histopathological Parameters in Endometrial Adenocarcinoma
Source: J Clin Med. 2026 May 16;15(10):3840. doi: 10.3390/jcm15103840 (PMC13207272; doi:10.3390/jcm15103840)
Supplement: Supplementary file 1 [file jcm-15-03840-s001.zip › jcm-4265525-supplementary.pdf]

Supplementary Materials

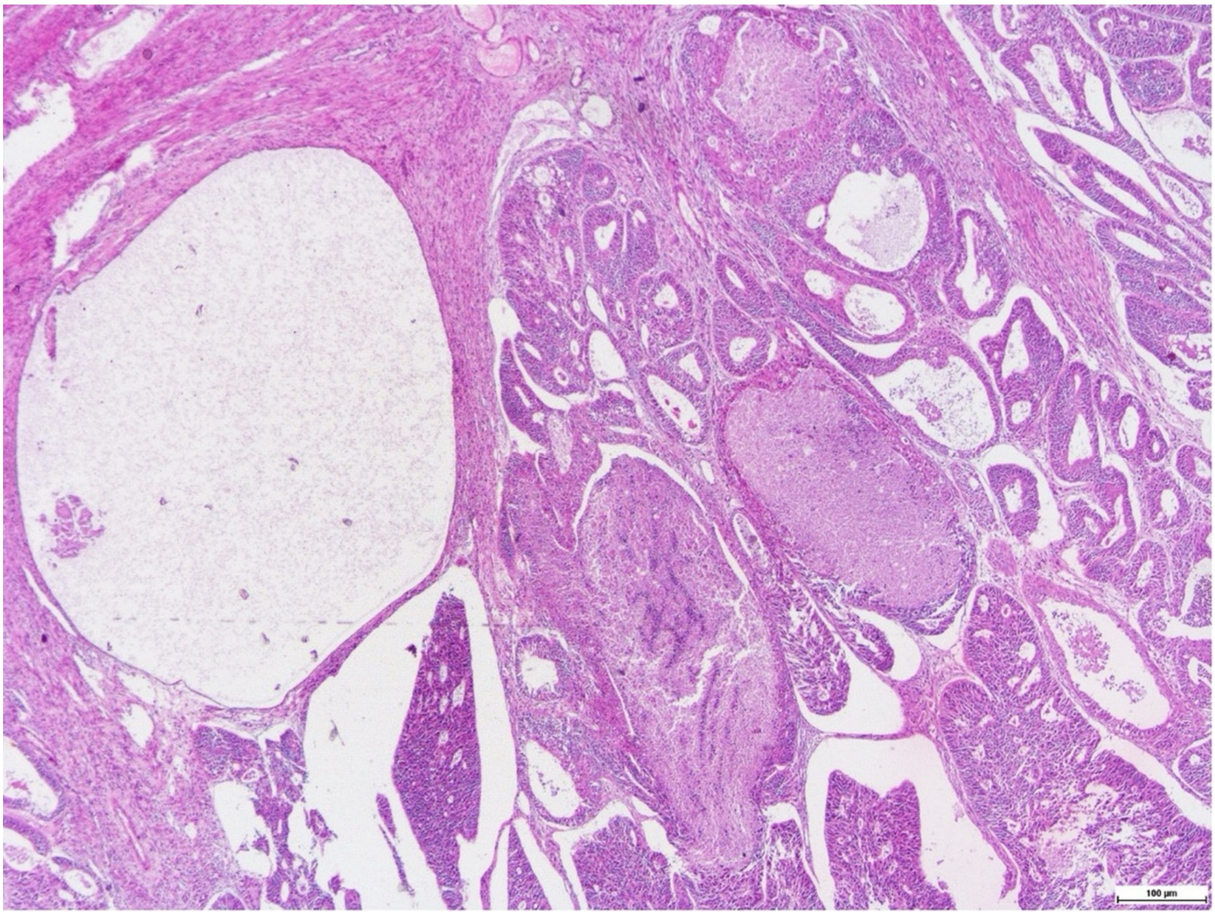

Figure S1. Typical endometrioid glands exhibiting a cribriform architecture within the stromal tissue (H&E ×100).

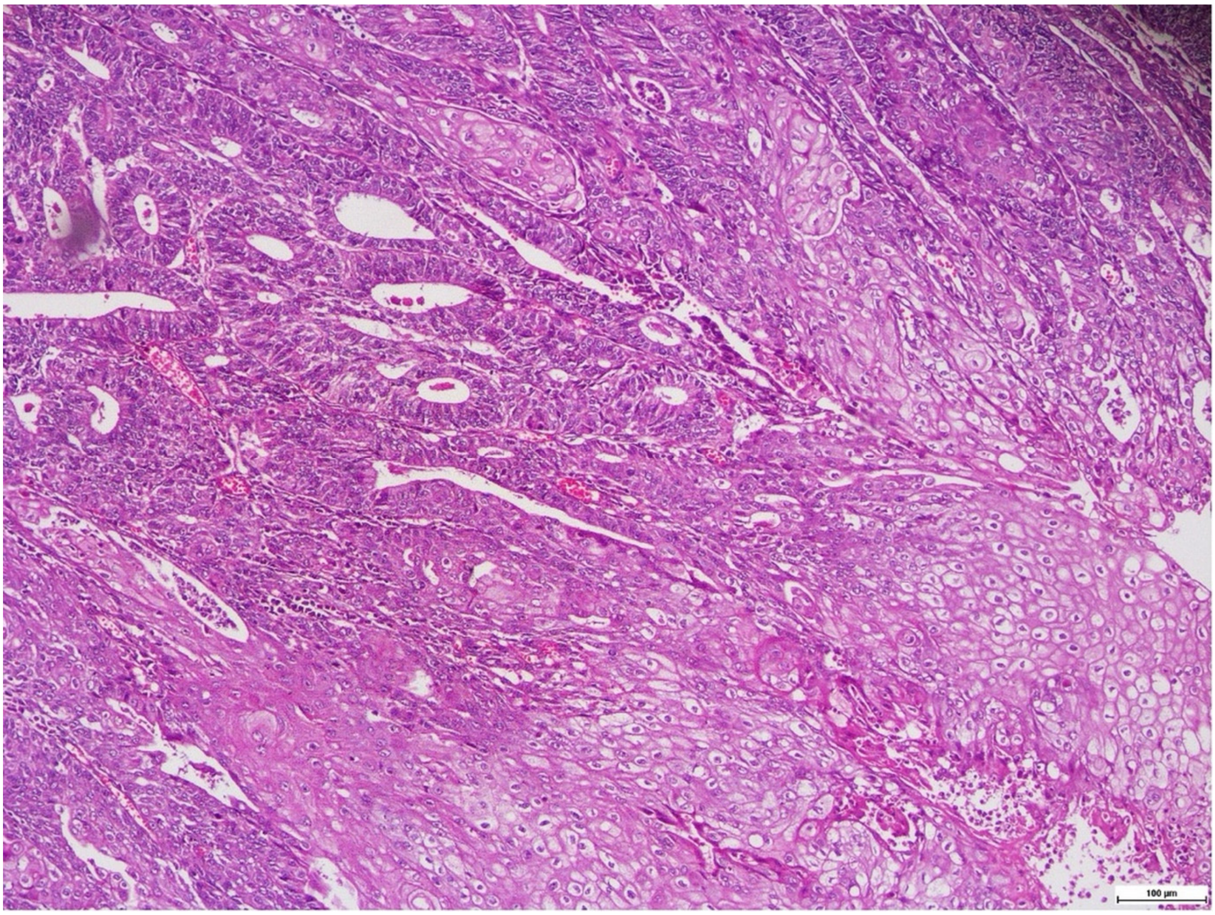

Figure S2. Cribriform glandular structures and squamous differentiation characteristic of endometrioid adenocarcinoma (H&E ×100).

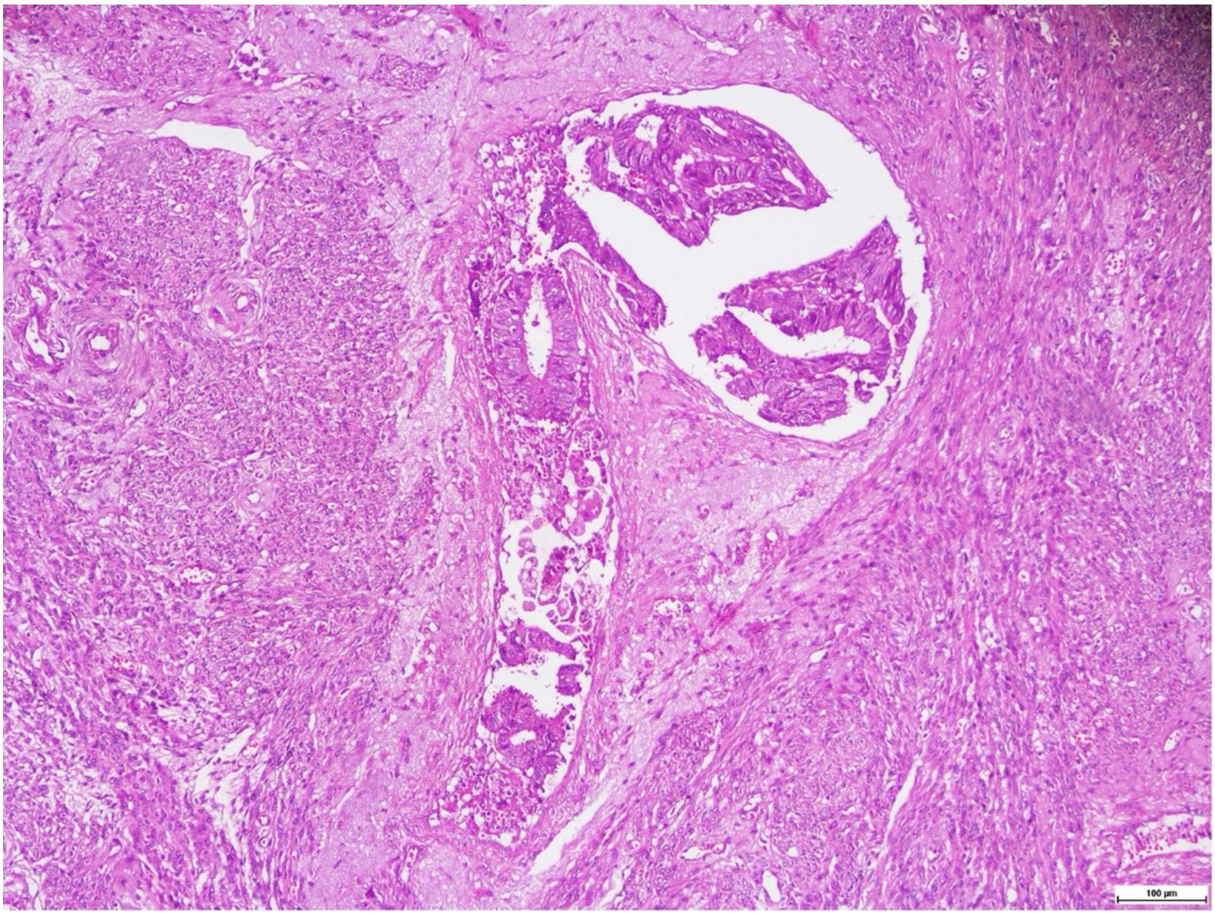

Figure S3. Lymphovascular invasion in endometrioid adenocarcinoma (H&E ×100).
